# Membrane Vesicles Derived from Gut Microbiota and Probiotics: Cutting-Edge Therapeutic Approaches for Multidrug-Resistant Superbugs Linked to Neurological Anomalies

**DOI:** 10.3390/pharmaceutics14112370

**Published:** 2022-11-03

**Authors:** Prakhar Srivastava, Kwang-sun Kim

**Affiliations:** Department of Chemistry and Chemistry Institute of Functional Materials, Pusan National University, Busan 46241, Korea

**Keywords:** blood–brain barrier, extracellular vesicles, gut microbiota, membrane vesicles, meningitis, probiotics, superbugs

## Abstract

Multidrug-resistant (MDR) superbugs can breach the blood–brain barrier (BBB), leading to a continuous barrage of pro-inflammatory modulators and induction of severe infection-related pathologies, including meningitis and brain abscess. Both broad-spectrum or species-specific antibiotics (β-lactamase inhibitors, polymyxins, vancomycin, meropenem, plazomicin, and sarecycline) and biocompatible poly (lactic-co-glycolic acid) (PLGA) nanoparticles have been used to treat these infections. However, new therapeutic platforms with a broad impact that do not exert off-target deleterious effects are needed. Membrane vesicles or extracellular vesicles (EVs) are lipid bilayer-enclosed particles with therapeutic potential owing to their ability to circumvent BBB constraints. Bacteria-derived EVs (bEVs) from gut microbiota are efficient transporters that can penetrate the central nervous system. In fact, bEVs can be remodeled via surface modification and CRISPR/Cas editing and, thus, represent a novel platform for conferring protection against infections breaching the BBB. Here, we discuss the latest scientific research related to gut microbiota- and probiotic-derived bEVs, and their therapeutic modifications, in terms of regulating neurotransmitters and inhibiting quorum sensing, for the treatment of neurodegenerative diseases, such as Parkinson’s and Alzheimer’s diseases. We also emphasize the benefits of probiotic-derived bEVs to human health and propose a novel direction for the development of innovative heterologous expression systems to combat BBB-crossing pathogens.

## 1. Introduction

The blood–brain barrier (BBB) plays a central role in the unique and complex microenvironment of the central nervous system (CNS) [1]. In particular, it restricts the entry of drugs and other exogenous molecules, including host immune cells [2] and infectious pathogens [3]. Nevertheless, opportunistic pathogens can occasionally breach the BBB and cause serious illnesses, including meningitis and brain abscess [4]. Although the occurrence of CNS infection is relatively rare, chronic malignancies can result in serious neurological disorders [5]. Drug-resistant pathogens, including *Acinetobacter baumannii*, *Escherichia coli*, *Listeria monocytogenes*, *Staphylococcus aureus*, and *Streptococcus* spp. can enter via the respiratory tract and mucosa and breach the BBB [6]. In the BBB, pathogens tightly regulate intrinsic virulence mechanisms via drug-resistance pumps [7] and biofilm formation [8]. *Acinetobacter* spp., *Klebsiella*, and *S. aureus* further modulate the expression of proinflammatory cytokines [9] and movement of immune cells, thereby destabilizing the endothelial lining and tight junctions of the BBB [10]. However, due to the complexity of the brain microenvironment and its associated endothelial tight junctions, transport of effective antimicrobials and therapies is challenging [11]. In fact, the physiological nature of the CNS environment prevents 90–95% of antimicrobials from progressing toward drug development [12]. Various nanoparticles (NPs), especially liposomal NPs [13,14], and their derivatives (e.g., polysaccharide and polyester NPs) [15,16] are considered effective and innovative drugs against pathogens that invade the BBB. However, NP-associated toxicity [17] and dose-dependent mortality [18] seriously limit their application. It is, therefore, necessary to consider alternatives, particularly those that can mimic non-immunogenic biological entities [19].

Membrane vesicles or extracellular vesicles (EVs) play crucial roles in polymicrobial interkingdom communication [20]. Microbial evolution involves the continuous transfer of metabolites via nanosized vesicles that carry important biomolecules, virulence factors, and membrane receptors of the cells from which they originate [21] to proximal and distant cells via blood and lymphatic systems. These vesicles range in size from 20 to 400 nm. The release of EVs is a general phenomenon performed by many cell types, including those of eukaryotes, Gram-negative/-positive bacteria, and archaea [22], as a means of communicating with other cells. In particular, bEVs have been characterized as the delivery vehicles of host–microbe interactions, responsible for the delivery of signaling molecules, such as autoinducers, virulence factors [23,24], and antibiotic genes [25,26]. In contrast to pathogen–host interactions, mucosal- or gut microbiota-derived bEVs contribute to homeostasis, immune system regulation, bowel movements, and the gut–brain axis [27]. Based on their immunomodulatory properties, gut microbiota-derived bEVs are currently employed in therapies aimed at promoting both humoral and cell-mediated responses [28]. Among them, tuning probiotic-derived bEVs, for interactions between interstitial cells and the gut–brain axis, represents a novel strategy for promoting immune responses during infectious disease [29]. Furthermore, this strategy can benefit from the ease of fermentation culture techniques, potential application of probiotics, and mucoadhesive encapsulation [30,31]. Moreover, combining functional biomaterials with active bEVs has the potential to target autoimmune inflammatory dispositions and treat severe chronic infections [32]. More specifically, beneficial gut microbiota-derived bEVs are a promising tool to regulate the gut–brain axis by reducing inflammation and restoring immunity [33], creating a benchmark for the targeted delivery of drugs to the CNS. However, currently, most EV-based drugs are derived from eukaryotic systems, including those for cancer [34], gastric disorders, and polymicrobial infections, due to the various challenges related to bEVs [35]. Nevertheless, genetically modifying bEVs via surface remodeling [36] to target neurotransmitters and quorum sensing (QS) inhibitors, and through CRISPR/Cas system-based modifications [37], has the potential to provide novel noninvasive therapies against BBB infections [38].

In this review, we introduce cutting-edge research on the mechanism of action and production of gut microbiota- and probiotic-derived bEVs against pathogens crossing the BBB. Hence, this article will serve as a valuable resource for future research aimed at enhancing the production of probiotic-derived bEVs in the context of antimicrobial research and designing novel heterologous expression systems.

## 2. Blood–Brain Barrier (BBB): A Roadblock to Invading Pathogens

The endothelial layer of the BBB selectively transports immune cells and other metabolites involved in maintaining the functional stability of the nervous system [39]. However, during the neonatal period, in some cases, the BBB can shield pathogens, resulting in a breach of the protective layer and subsequent serious disorders and infection [40]. Endogenous markers, such as pathogen-associated molecular patterns and small molecular motifs conserved within a class of microbes [41], are recognized by endothelial receptors of the BBB. This recognition results in an immunological burst at the target site [42] that can breach the endothelial lining. Moreover, the complicated structure of the CNS limits the access of several antimicrobial agents to the nervous system [43], however, facilitating the transport of lipophilic drugs with a molecular weight <400 Da [44] that form fewer than eight hydrogen bonds via lipid-mediated free diffusion [45], into the bloodstream via the transcellular route [46]. As efficient drugs, antiepileptics (e.g., diazepam and phenytoin) [47,48], PLGA-coated nanoparticles, and laser-assisted therapies (e.g., focused ultrasound and interstitial thermal therapy) are commercially available [49,50]. However, these therapies do not guarantee the long-term potency of drugs because the microbial flora is constantly evolving, either through horizontal gene transfer or cell-to-cell communication, resulting in reduced susceptibility to certain drugs [51].

## 3. Multidrug-Resistant (MDR) Superbugs: A Prominent Case Involving the BBB

Infections caused by MDR superbugs have emerged as a major threat to global health in the post-antibiotic era, especially in the 21st century [52]. The Centre for Disease Control and World Health Organization have predicted that there will be ~2 million cases of MDR infections and 27,000 related deaths per year by 2050 in Asia, Africa, and North America [53]. Carbapenem and colistin are the most widely used last-resort antibiotics against bacterial infections [54]; however, by the late 2000s, drug resistance exhibited an unexpected increase in mortality associated with hospital-acquired infections by 40–60% [55]. Pan-drug resistant *A. baumannii* is routinely reported in patients with meningitis [56,57,58] and has acquired resistance to most antibiotic therapies, including colistin and tetracycline [59,60]. Although combined treatment with gentamicin and meropenem is efficient, the reduction rate of infection is <17–19% [61] given that the BBB limits the permeability of drugs and the continuous administration of drugs further increases the probability of resistance [62,63]. Moreover, frequently screened drug-resistant pathogens (*A. baumannii* [64] and *E. coli* [65]), few routinely screened pathogens (*N. meningitidis* and *Streptococcus* spp.) [66,67], other neuroinvasive pathogens (*Haemophilus influenzae*) [68], and *Chlamydophila pneumoniae* [69] not only disrupt the tight junctions of the BBB but also induce leakage between tight junctions and vascular endothelial cells [70]. For example, Gram-positive *L. monocytogenes*, *Staphylococcus* spp., and *Streptococcus pneumoniae* elevate the levels of proinflammatory cytokines and disrupt the endothelial lining in the CNS [71], thus creating a path of invasion for opportunistic pathogens.

## 4. Bacteria-Derived EVs (bEVs): Nanoscale Vesicles

bEVs have been studied since the early 1960s when lipid-like structures released from *E. coli* were discovered as a means to transport secondary metabolites and intrinsic biomolecules to the communicating host [72]. After the discovery of bEV production from Gram-positive bacteria, such as *Bacillus subtilis*, *Mycobacterium tuberculosis*, *S. aureus*, and *Streptococcus* spp., bEV release is regarded as a general phenomenon carried out by bacteria that has an important role in cell-to-cell communication and disease progression during gastric cancers and tuberculosis [73,74]. Cell-to-cell communication by bEVs involves internalization via the endothelial layer, micropinocytosis, and endocytosis by utilizing invasion proteins at the host–pathogen interface [75]. Certain pathways, such as the stress induced network, cause bEVs to function as anti-phagocytosis bodies, evading phagocytosis and weakening the clearing mechanism via the host immune response [76]. *M. tuberculosis* is a classic example of pathogen evasion of the innate immune responses; that is, it infects phagocytic and inhibits phagosome maturation. Moreover, Athman et al. [77] discovered that *Mycobacterium* bEVs produce lipoglycans and lipoproteins that play an important role in regulating the host immune response and facilitating persistent infection. Further, it was found that *S. aureus*-derived bEVs contain super-antigens (protein A and lipase) that aid cells in phagocytosis evasion. Meanwhile, a proteomics study [78] found that immunoglobulin (IgG)-bound lipase and super-antigen (hydrogenated form of squalene; SQA) are presented in bEVs, thus highlighting the potential role of *S. aureus* in evading anti-phagocytic activity via super-antigens and lipase production. bEVs also have a basic role in exchange of genetic materials (DNA and RNA) through horizontal gene transfer, during which bEVs serve as a means of cell-to-cell communication within the same bacterial species [79]. Additionally, a study conducted on bEV cargo of *A. baumannii* reported the presence of a carbapenamse gene (*bla*_OXA-24_) that increases the antibiotic susceptibility pattern against β-lactam antibiotics [80]. Similar studies on bEVs derived from *N. gonorrhoeae* [81] and *S. aureus* [82] have identified the presence of the outer membrane (OM) protein PorB and alpha toxins that transfer genetic materials, inducing apoptosis and host cell death.

bEVs released from the cell envelope of Gram-negative bacteria are so-called outer membrane vesicles (OMVs). The envelope is made up of three layers: the OM, cytoplasmic membrane, and the periplasmic space in between, which contains a layer of peptidoglycan (PG) [83]. An inside leaflet of phospholipids and an outer leaflet of lipopolysaccharide (LPS; also known as endotoxin) constitute the OM. LPS causes inflammatory responses in host cells [84], whereas the OM has a porous structure that aids in waste removal and nutrition uptake, and the peptidoglycan (PG) layer maintains the osmotic pressure of the cell and regulates the hostile environment (antibiotic stress) [85]. Gram-positive, unlike Gram-negative, bEVs are produced from cytoplasmic constituents via a blebbing mechanism; their genetic composition is comparable to that of Gram-negative bEVs, with the exception of the lipoprotein structure [86]. Apart from the normal mechanism of blebbing, prophage-encoded endolysins have also facilitated bEV release from Gram-negative and -positive bacteria. Studies on *Bacillus* spp. and *Staphylococcus* spp. have revealed that the prophage-encoded endolysin generates holes in the peptidoglycan cell wall, thus highlighting the potential role of these enzymes in bacterial cell wall lysis during mass production of bEVs [87,88].

## 5. Nanoscale bEVs as Potential Therapeutic Platforms

Recently, bioinspired NPs such as host (eukaryotic) EVs (hEV) and bEVs have shown promising effects against chronic infections [89,90]. Compared with their nanomaterial counterparts (liposomal NPs), bEVs provide increased drug delivery and efficient antigen-presenting properties [91,92,93]. Various microbes including *Helicobacter* spp., *Klebsiella pneumoniae*, *Lactobacillus* spp., *P. aeruginosa*, *S. aureus*, and *Streptococcus* spp. are involved in the transfer of metabolites between species for intracellular communication and are used in novel adjuvant-associated therapeutics as well as nano-sized vaccine delivery platforms for various infections [94,95] (Table 1).

hEVs have shown complexity of the yield coefficient, a high production cost, and limited downstream process, all of which limit their biomedical applications [110,111] (Table 2). The continuous evaluation of EVs as potential tools against chronic infection has led to the development of bEVs derived from *Clostridium butyricum* [112] and *L. paracasei* [113]. Given that most chronic illnesses involve ‘dysbiosis’ of the gut microbiota, tuning the absorption capacity and nutrition digestion factors of the microbiome might influence the host–microbe physiological imbalance.

## 6. Unresolved Issues with Gut Microbiota-Derived bEVs in Modulating the Gut–Brain Axis: *Old Is Gold*

The continuous usage of antibiotics during BBB infections leads to prognosis of early psychosis and neurotoxicity [114]. Gut microbiota dysbiosis, a state where the physiological combinations of flora are transformed into pathological combinations [115] via continuous antibiotic administration, has been linked to neural abnormalities. This link is via the vagal nerve, which is associated with a lower response of neurotransmitters inducing systemic inflammation in the CNS [116]. These features highlight the importance of the gut–brain axis in modulating CNS homeostasis.

### 6.1. Gut–Brain Axis

The term ‘*Gut–Brain axis*’ refers to a bidirectional network that includes multiple connections such as the vagus nerve (nervous control), immune coordination (epithelial and mucosal barrier), and secondary metabolite generation from microbes [117]. The complex architecture of the gut–brain axis entails the constant transit of neurotransmitters within the gastrointestinal (GI) tract, which, in turn, modulates the immune system, including macrophages and mast cells [118]. These immune cells boost neuron excitability and regulate the host’s behavioral response. A recent study found that gut dysbiosis caused by a broad-spectrum antibiotic during traumatic brain injury (TBI) resulted in increased neuronal loss, suppressed neurogenesis, altered microglia and peripheral immune response, and modulated fear memory response, suggesting a role of gut microbiota in the recovery from TBI [119].

### 6.2. Gut Microbiota-Derived bEVs vs. Eukaryotic-Derived hEVs (Physiological Counterpart)

Generally, the use of hEVs is significantly limited by the yield coefficient and high-throughput screening. In addition, the current scenario for combating antibiotic resistance with chronic illness is not favored by the use of pathogen-derived bEVs, because the sudden release of pro-inflammatory factors by bacteria cannot be controlled [120]. In contrast, beneficial gut microbiota have shown the effective immune responses and efficient pathogen inhibition activity [121]. Moreover, bEVs from beneficial gut microbiota take a role in triggering inflammatory responses through LPS and lipoteichoic acid [122] and can cross the intestinal barrier, and have effective anti-inflammatory properties against chronic infections and gut dysbiosis [123]. The physiological features of hEVs differ significantly from gut microbiota-derived bEVs, as shown in Table 3.

### 6.3. Problems Related to Gut Microbiota-Derived bEVs on BBB-Associated Diseases

The ‘dysbiosis’ condition in the gut microbiota environment by antibiotic usage has also shown certain detrimental impacts such as Alzheimer’s disease, autism, and arthritis, all of which clearly demonstrate the mechanistic behavior and coordinated axis of mental health and intestinal mucosa [127]. A study by Lee et al. [128] showed that the release of bEVs from a gut pathogen *Paenalcaligenes hominis*, revealed movement of bEVs via the vagus nerve, producing cognitive impairment in the nervous system. Another study using *Porphyromonas gingivalis*, an oral pathogen, demonstrated the importance of LPS-coated bEVs in the onset of Alzheimer’s disease, emphasizing the role of protease and LPS in triggering the damage of collagen fibers, fibrinogen connective tissues, and induction of proinflammatory mediators in the transfer of bEVs that alter brain cognitive function [129]. The main drawback of bEVs derived from the gut microbiota is that they have a negative impact on memory, cognition, and neuroinflammation. Therefore, direct application of such bEVs may have both adverse and beneficial neurologic effects on CNS homeostasis.

### 6.4. Beneficial Roles of Probiotic-Derived bEVs on Gut–Brain-Axis Control

Considering the diverse array of gut microbiota from intestinal niches, probiotics including *Bifidobacterium* spp. and *Lactobacillus* spp. have been identified to create neurotransmitters (acetylcholine, gamma-aminobutyric acid (GABA), and serotonin), which continually control CNS homeostasis [130,131]. Overall, probiotics not only govern the bidirectional transit of biochemical signals, but also improve the host’s behavioral response such as anxiety [132], depression, and stroke [133]. Apart from periodontal and gut pathogens, probiotics such as *Lactobacillus* spp. have influence on the motor neuron complex (M-N complex). This M-N complex includes the enteric nervous system (endocrine functions and secretion from intestinal mucosa) and the vagus nerve. *Lactobacillus* spp. normally modulates the neurotransmitter signals via the vagus nerve (intestinal nerve), involving sensory transmission of neuronal signals via the enteric nervous system to the CNS [131]. Few bEVs derived from *Lactobacillus* spp. have also demonstrated the direct regulation of the gut–brain axis in CNS homeostasis (Table 4).

## 7. Filling Gaps with Probiotic-Derived bEVs against BBB-Breaching Pathogens

The fundamental issue with antibiotic therapy is the associated drug resistance, which causes a widespread distribution of MDR BBB-breaching bacteria, causing secondary neurological disorders that impair CNS homeostasis [138]. Moreover, the risks associated with the continuous use of antibiotics include seizure, neuromuscular blockade, cranial nerve toxicity, and intracranial hypertension [139]. Conventional therapeutic options for the treatment of MDR bacterial infections include β-lactamase inhibitors, aminoglycosides, fluoroquinolones, and last-resort polymyxins [140,141]. However, such therapies have limited efficacy in CNS infections caused by MDR bacteria, such as *A. baumannii*, *K. pneumoniae*, *M. tuberculosis*, *L. monocytogenes*, *N. meningitidis*, and *Streptococcus* spp. (Table 5). This is due to the BBB integrity as well as severe side-effects such as neurotoxicity and nonspecific targeting. The continuous administration of antibiotics, and its associated risk factors, often creates dysregulation between the gut microbiota and the cerebrospinal fluid of the CNS. Mucosal bacteria regulate the communication between the enteric nervous system and peripheral intestinal regulation. Meanwhile, the constant dysregulation caused by antibiotic overuse has created a gap between efficient metabolism of intestinal regulation and CNS modulation.

Several probiotics such as *Bifidobacterium* spp., *L. lactis*, and *L. rhamanosus* are being actively investigated for their therapeutic potential and are in the final stage of clinical trials [155]. bEVs originated from such species have gained attention as effective therapeutic platforms owing to their natural immunogenicity and self-adjuvating properties [156], which induce a better adaptive immune response and can transport diverse cargos across various cell types. In addition, such therapeutic platforms against antibiotic-resistance-related neurological disorders could be improved using genetic modification of gut microbiota with the CRISPR/Cas9 system [157]. The antimicrobial activity of probiotic-derived bEVs against pathogens has revealed a broader role of probiotics in enhancing the anti-inflammatory response during pathogen invasion (Table 6).

A proteomics study of probiotic *L. plantarum* BGAN8-derived bEVs that regulate brain function [136] revealed the enrichment in enzymes involved in central metabolic pathways and in membrane components with transporters [162]. Because such proteins are associated with transferring beneficial metabolites to pathogens or hosts, proteomics of probiotic-derived bEVs appears to be a potential tool to reveal underlying mechanisms of bEVs on escaping pathogen infection and the beneficial effect on brain function.

**Figure 1:**
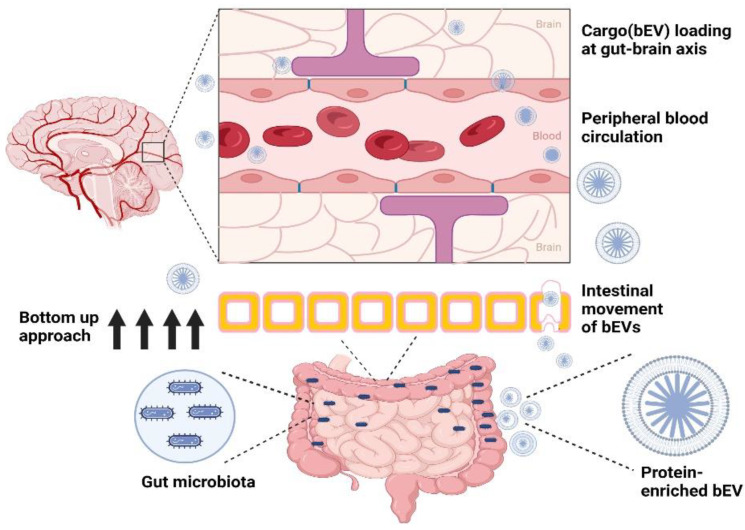
Bottom-up approach for the transport of probiotic-derived extracellular vesicles (bEVs). The figure was created using BioRender.com (https://app.biorender.com; accessed on 2 November 2022).

Vaccine-antigen-presenting probiotic-derived bEVs can also be employed as vehicles to transport antigens and potent antimicrobial agents to specific targets. Moreover, given that probiotics regulate various intrinsic signals, such as regulation of active short chain fatty acids, hormone metabolism, and neurotransmitters signaling and expression, they can also facilitate a wide range of interactions between the normal flora and host cognitive behavior [163]. Moreover, their role in regulating the gut–brain axis has highlighted the potential application of probiotic-derived bEVs for enhancing the neurodevelopment process [164]. For instance, studies with *L. plantarum* JB-1-derived bEVs highlighted the role of bEVs in regulating the neuron signaling system [165], demonstrating the direct role that probiotic-derived bEVs have in CNS development [166] (Figure 1). Moreover, unlike host immune cells, bEVs derived from immune cells can pass though the BBB and, thus, participate in the immunological regulation of the CNS.

## 8. Remodeling of Probiotic-Derived bEVs against BBB-Invading Pathogens

NP-derived therapeutics show high efficacy against pathogens [167]. As it is possible to control the size and release of NP-derived therapeutics, modified NPs are potential candidates for conferring protection against drug-resistant pathogens [168]. Combination therapies with commercial antibiotics also exert beneficial effects against antibiotic-resistant pathogens; however, their selectivity and toxicity remain major concerns [169]. The modification of NPs with EVs has been driven by the aim to increase yield and reduce toxicity [170,171]. For instance, amalgamated nanocarriers with hEVs have shown promising results in cancer therapy [172]. However, to induce an efficient immunogenic response with low toxicity, bEVs from probiotics should be used in native or genetically modified form to protect against hospital-acquired infections [173]. Such an engineered, or remodeled, probiotic-derived bEV will have advantages over conventional drug delivery systems in terms of their bioavailability and targeted drug distribution.

### 8.1. Surface-Modified Proteins in Probiotic-Derived bEVs

Exosome-associated transmembrane proteins and their fusion to peptide domains have been investigated for their ability to confer protection against various pathological conditions in eukaryotes. For example, their role in tumor therapy, and the delivery of siRNAs and miRNAs targeting immune cells and neuronal junctions of the brain, have been evaluated [174]. However, targeting the efficiency of surface-modified probiotic-derived bEVs has not been evaluated in infections involving BBB breach. Among prokaryotes, a two-component signaling system in Gram-positive bacteria regulates diverse intracellular signals, including genetic transduction and bacteriocin production [175]. Studies conducted on *Bifidobacterium* spp., *L. gasseri*, and *L. plantarum* have demonstrated the role of the two-component system in the regulation of bacteriocin production [176]. For instance, surface-associated proteins of *Lactobacillus* spp., such as histidine protein kinase (HPK), and S-layer proteins (SlpA*,* B, and X) [177], have been explored in bEV studies for evaluating heterologous gene expression and enhancement of host–microbe interactions. HPK-associated recombinant protein expression is a novel approach for biotherapeutic delivery. Previous studies on bEVs revealed that the expression of heterologous antigens such as OmpA was in response to infection severity. The production of fusion proteins and hemolysin ClyA in *E. coli* bEVs elicited an immune response against green fluorescent (GFP) protein [178]. The concept may involve the association of a signal peptide with a reporter system that can trigger the robust secretion of target molecules for cell surface display. Similarly, the Slp system has been studied extensively in *L. acidophilus* and *L. brevis* against diarrhea and skin infections [179]. A system with strong transcription facilitated by promoter fusion [180] could increase protein production and provide a useful vaccine delivery platform. Strategies for protection against infections crossing the BBB may involve the addition of the Slp short peptide region to the upstream region of the targeted antigen or therapeutic gene, which can increase the secretion and efficacy of the therapeutic protein against infectious agents [181].

### 8.2. Regulation of Neurotransmitters across the BBB

BBB-associated infections are related to Parkinson’s and Alzheimer’s diseases, in which direct correlations between pathogens such as *Staphylococcus* spp. have been demonstrated to regulate the neurotransmitter-serotonin signaling mechanism [182]. To date, the interaction of probiotics with host miRNAs in regulating host cerebral inflammatory signaling is rare. Instead, the regulation of the bidirectional movement of miRNA by probiotic-derived bEVs in regulating the neuro-immune endocrine regulation was reported [183].

Only a few studies examining the relationship between probiotics and the serotonergic system, as well as the role of the GI tract in managing neuropsychotic disorders, have been reported. For instance, a previous study [30] reported that *Akkermansia muciniphila*, an intestinal symbiont colonizing the mucosal layer, increases the serotonin signaling pathway via the gut–brain axis in mice. More specifically, they showed that downregulation of *Htr* mediators (secreted metabolites in the colon) in the intestinal mucosa activates the bacterial colonization and, hence, increases the serotonin level and enteric neuronal activity. One classic study on probiotic supplements, including short-chain and long-chain oligosaccharides, showed that lower expression of *Htr* reduces anxiety behavior in mice, thus demonstrating the possible significance of probiotics in maintaining neurotransmitter signaling [184].

### 8.3. Quorum-Quenching Proteins

Studies of microbiome-associated neurological disorders have supported the systemic movement of quorum-sensing molecules [185] and their associated virulence factors. These factors penetrate tight junctions using the Trojan horse method and trigger nervous system connections [186]. Pathogens (e.g., *Clostridium* and *Streptomyces* species) can induce nervous system dysregulation and result in anxiety and stress-associated disorders [187]. The association between polymicrobial infections and common neurological disorders has been clarified; however, well-established tools to overcome chronic-infection-associated neurological disorders, such as bacterial meningitis and polymicrobial-associated multiple sclerosis, are needed [185]. Most neurological disorders are accompanied by a decreased abundance of beneficial, as well as commensal, microbes. Accordingly, it may be possible to express quorum-quenching-related proteins on the surface of probiotic-derived bEVs, as a targeted approach against microbes to reduce their virulence and chronicity.

### 8.4. bEVs as a Drug Delivery Platform to Prevent Degradation and Immune Elimination of Antimicrobials

Regarding CNS infections, most CNS-associated drugs have side-effects and lack the potential to cross the BBB. Additionally, the inefficient movement of neurotherapeutic drugs requires them to remain in the neural environment for a sufficient duration to exert the desired effect. The presence of phosphorylated glycoprotein (P-gp) in the endothelial lining of the BBB undoubtedly limits the entry of lipophilic drugs, thus increasing the risk of meningococcal infections [188]. Antibiotics such as vancomycin, meropenem, fluoroquinolones, β-lactams (occasionally), and cephalosporins are thought to be effective against CNS infection. However, inefficient administration and toxicity levels limit their usage [189]. In this case, bEVs can act as high specific loading cargos for antibiotics to provide a shielding effect [190], which will protect the antibiotics against various pathogen-derived enzymes and multi-antigen determinants on the surface will specifically target meningococcal infections [191].

### 8.5. CRISPR/Cas-Modified bEVs as Biotherapeutic Agents against BBB-Breach-Related Infections

**Figure 2:**
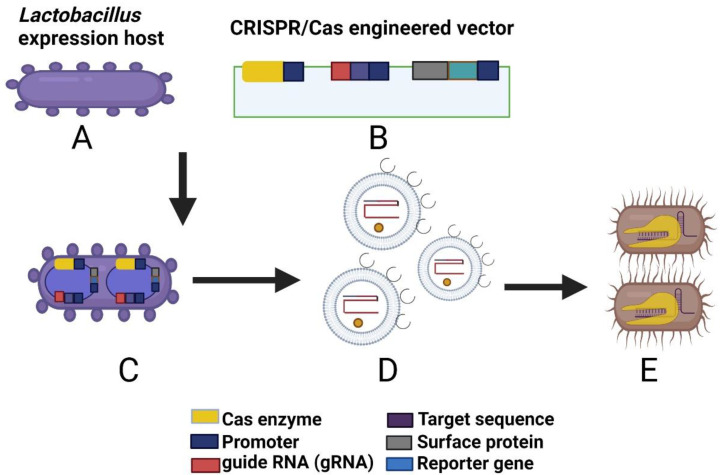
Application of the CRISPR/Cas system for the development of biotherapeutic tools against infections crossing the BBB. (**A**) *Lactobacillus* expression host, (**B**) CRISPR/Cas expression vector, (**C**) expression of the engineered vector in *Lactobacillus*, (**D**) CRISPR/Cas-enriched *Lactobacillus* bEV, and (**E**) targeted therapy against pathogenic bacteria via surface protein receptors. The figure was created using BioRender.com (https://app.biorender.com; accessed on 2 November 2022).

The CRISPR/Cas system is a novel gene editing approach that has been successfully employed to make opportunistic pathogens (e.g., *E. coli* and *S. aureus*) vulnerable to commercially available antibiotics, or to reverse their drug resistance [192] (Figure 2).

This approach involves identification of the Cas system from *Lactobacillus* species (Type I or Type II system), constructing an engineered vector model and designing an expression system based on the surface modification of a targeted ligand using a reporter system [191] comprising an inducible promoter sequence, guide RNA, Cas9, selectable marker, surface protein with a reporter gene, and target DNA sequence. Using this approach, the entire vector can be transformed into the *Lactobacillus* via electroporation or microfluidic injection. bEVs from transformed *Lactobacillus* contain surface-expressed heterologous proteins that can be targeted to the specific host cell receptors for vaccine therapy. Studies on *L. reuteri* and *L. sakei* [193,194] have identified the presence of 20–25 CRISPR systems with varying degrees of polymorphism, conferring an evolutionary advantage against invasive pathogens. Recent examples of *L. acidophilus* and *L. crispatus* delivery mechanisms using the Slp system (S-layer membrane protein) (see Section 8.1) [180] have highlighted the utility of genome editing tools in probiotic species. Therefore, the CRISPR/Cas system can facilitate development of tools targeting drug-resistant pathogens and create avenues for designing potent and targeted therapeutic strategies against infections crossing the BBB.

**Figure 3:**
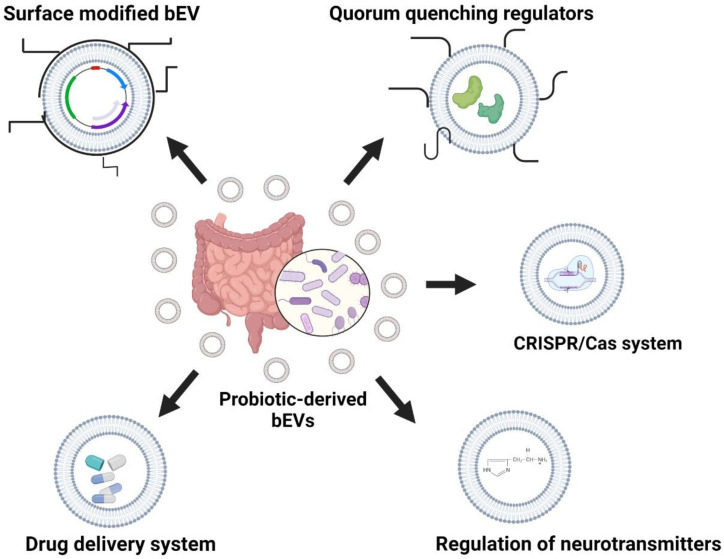
Remodeling of probiotic-derived bEVs against BBB related anomalies. The figure was created using BioRender.com (https://app.biorender.com; accessed on 2 November 2022).

The potential role of probiotic-derived bEVs can provide numerous benefits against hospital-acquired infections. The fine tuning of probiotic-derived bEVs on parameters such as quorum-quenching enzymes and the CRISPR/Cas mechanism can provide a possible strategy to target secondary risk factors associated with dysbiosis in the gut–brain axis (Figure 3).

## 9. Potential for Application of Probiotic-Derived bEV Platforms against BBB-Associated CNS Infections

Infections with MDR bacteria, which secrete various virulence factors and toxic proteins that target sensitive regions of the brain, can readily cross the BBB’s endothelial barrier and cause serious neurological disorders. Furthermore, the robust movement of various immune cells at the site of injury promotes localized inflammatory responses and results in cytokine bursts, thus affecting CNS permeability and causing neurological imbalance.

Probiotic-derived bEVs represent safe therapeutic agents against a variety of infections and outperform conventional antibiotic therapy for BBB-associated CNS infections. However, the efficacy of bEVs derived from probiotics other than *L. paracasei*, in the treatment of CNS infections, is currently under evaluation in ongoing clinical trials [195]. Indeed, the presence of CNS inflammation can significantly impact bEV efficacy as it reduces the amount of drug crossing the CNS barrier, which is impeded by BBB-mediated exclusion. Nevertheless, certain drugs, including citalopram, doxepin, erythropoietin, and fluvoxamine, have demonstrated significant anti-neural anomaly activity [196]. However, these drugs are limited by their low membrane permeability, rapid clearance, and rapid degradation. Therefore, additional treatments are now being developed, such as nano-based drug delivery agents, liposomal NPs, and biomimetic NPs or nanocomposites with the potential to penetrate the BBB. However, studies using anti-seizure drug-loaded gold NPs revealed increased oxidative stress [195], necessitating a re-evaluation of the associated dosing regimen. Similarly, chitosan-based NPs exhibit minimal BBB absorption and are not currently used in clinical practice [196]. Meanwhile, for extended periods of usage, liposomal NPs outperformed metallic counterparts in post-stroke inflammatory responses. However, their instability, shorter lifetime, and restricted drug encapsulation capability limit their use as a drug delivery vehicle for nondegenerative disorders.

In contrast, bEVs outperform lipophilic and hydrophilic/hydrophobic drugs. In fact, a bEV derived from *Chromobacterium violaceum—*a facultative anaerobic, oxidase-positive, glucose-fermenting, non-lactose-fermenting, Gram-negative *Bacillus—*was successfully used to encapsulate violacein by enhancing its absorption coefficient [197]. Hence, due to their direct linkage with the gut–brain axis, as well as their movement via the autonomic nervous system, bEVs might represent an alternative drug-encapsulating vehicle for treatment of BBB infections; however, it is necessary to first address the issues regarding their bioavailability and surface modifications. In fact, probiotic-derived bEVs represent a useful platform for the development of new treatments as they have been shown to improve immunogenic responses to numerous pathogens that affect the gut–brain-axis function.

Genetic engineering of hEVs has recently been recognized as a paradigm shift in the treatment of CNS infections. Therefore, modified eukaryotic hEVs are regarded as effective delivery vehicles for hydrophobic and hydrophilic medicines. However, improving the ability of hEVs to invade the BBB has proven challenging. In this regard, probiotics with enhanced invading BBB activity might be viable therapeutic options against BBB-associated MDR pathogen infections. Most microbiota-related neurological disorders are associated with an imbalance of intestinal commensal bacteria, and probiotic-derived bEV-based platforms provide a successful therapy against CNS infections.

## 10. Future Research and Perspective

Strong efforts are required to improve the design of therapeutic agents that target MDR superbugs associated with the BBB. Unlike eukaryotic hEV biomarkers, proteins of probiotic-derived bEVs remain unidentified, thus limiting the utility of bEVs in BBB-associated therapy. Therefore, multiple omics approaches and in silico analysis are warranted. Additional high-throughput-scale functional analysis is required to identify potential therapeutic proteins of bEVs and design novel platforms for the selective and efficient targeting of BBB-associated infections that also elicit memory T cell responses to establish long-term immunity. Notably, most probiotic-derived bEVs exhibit antibacterial activity and enrichment of antibacterial metabolites. Thus, probiotic-derived bEVs can be used in combination with commercial antibiotics or repurposed drugs to increase their therapeutic efficacy against pathogens. With the aid of cheminformatics [197], formulated antimicrobial analogs can be designed to target pathogenic microbial factors. Indeed, this approach is expected to expand the current scope of antimicrobial use by generating probiotic-derived bEVs to effectively treat BBB-breaching infections.

## 11. Conclusions

The recent literature has demonstrated the effectiveness of EVs against various infectious pathogens. However, most research has largely focused on developing therapeutics or drug delivery vehicles by utilizing either NPs or hEVs (exosomes). Although these agents are clinically significant, their utilization is limited by long-term toxicity and the related mortality, low immunogenic response, stability issues, cost of scaling up, fermentation culture conditions, and downstream processing. In contrast to hEVs, there are only a few FDA-approved therapeutic bEVs, including a bEV vaccine (MeNZB) cleared for use against *N. meningitidis.* This is due to either failed trials or a low therapeutic efficiency. The concept of ‘postbiotics’ has recently been evaluated as a source of nonviable bacterial supplements capable of regulating the gut–brain axis. That is, the use of probiotics alone may be limited in scope; however, it can be enhanced by tuning the active components of postbiotics to initiate the release of probiotics-derived bEVs or -enriched bEVs. Meanwhile, limitations of combining NPs with antimicrobial compounds have hampered their application for the treatment of infections; moreover, this strategy does not address safety issues related to BBB breach. Collectively, the work summarized in this review provides insights into the efficacy of probiotic-derived bEVs and the novel concept of ‘postbiotics’ as a potential tool for the development of therapeutic platforms to overcome drug resistance in pathogens causing neurological disorders.

## Figures and Tables

**Table 1:** bEVs involved in the pathogenic infections and their roles.

Origin of bEV	Infecting Pathogens	Role of bEV	Reference
Gram-positive bacteria			
*Bifidobacterium longum*	Food-borne infections	Induction of progenitor cells	[96]
*Burkholderia* spp.	Activity against *A. baumannii* and *S. aureus*	N.D.	[97]
*L. gasseri*	Human Immunodeficiency Virus (HIV)	Change in susceptibility pattern of viral infection by regulation of toll-like receptor (TLR)-2 signaling	[98]
*L. rhamnosus*	Superficial infections	M2 Macrophage	[99]
*S. aureus*	*Pneumococcal* infection	TH1-mediated cell immunity	[100]
*Streptococcus* spp.	*Streptococcal* infection	Induction of dendritic cells	[101]
*Tetragenococcus halophilus*	Opportunistic pathogens	Anti-inflammatory factor interferon beta (IFN-β)	[102]
Gram-negative bacteria			
*Acinetobacter* spp.	Pan-drug resistant *A. baumannii*	Activation of IgG and IgM	[103]
*Borrelia burgdorferi*	*B. burgdorferi* colonization	Stabilizing superoxide	[104]
*Helicobacter pylori*	*H. pylori* infection	Induction of TH2 immune cells	[105]
*K. pneumoniae*	*K. pneumoniae* infection	Humoral and cellular immunity	[106]
*N. meningitis*	Meningococcal disease	IgG-mediated response	[107]
*Pertussis A*	*Bordetella pertussis* infection	Induction of CD4 cells	[108]
*P. aeruginosa*	Lethal dose of *P. aeruginosa*	Mixed cellular response	[109]

**Table 2:** Current limitations of eukaryotic and bacterial EVs in biomedical applications.

Eukaryotic (hEVs)	Bacterial (bEVs)	Common Limitations
Differentiation between cell surface markers	Lipopolysaccharide (LPS) toxicity	Immunomodulators outburst
Inefficient purification of vesicles	High inflammatory responses	Low viability and inefficient growth conditions
Lack of heterogeneity	High chance of infection (pathogen-derived bEVs)	High cellular toxicity

**Table 3:** Difference between eukaryotic-derived hEVs and gut-microbiota-derived bEVs.

Category	Eukaryotic-Derived hEVs	Gut-Microbiota-Derived bEVs
Biogenesis	Generally produced from plasma membrane except exosomes, which originate from endocytic pathway	Gram-negative bacteria: decreased protein linkages between the OM and peptidoglycan, accumulation of unfolded proteins and/or peptidoglycan in the periplasmic space, and explosive cell lysis Gram-positive bacteria: turgor pressure by accumulation of bEVs and the action of cell-wall-degrading enzymes
Composition (Cargo)	Multivesicular bodies composed of endosomal proteins; RNA and miRNA are regularly incorporated	Proteins, peptidoglycans, lipids, LPS, lipoteichoic acids (LTA), nucleic acids, and metabolites
Major functions	Intercellular communications (cell proliferation, matrix formation, and phagocytosis)	Innate and adaptive immunity, bacterial communications, interaction with host miRNA for movement across intestinal barrier
Size	40–100 nm (exosomes) [124]; 500–2000 nm (apoptotic bodies) and 100–500 nm (microvesicles) [125]	10–300 nm [126]

**Table 4:** Beneficial roles of probiotic-derived bEVs on gut–brain-axis control.

Origin of bEVs	Roles of bEVs	References
*L. acidophilus*	Changes in complex microbial communities	[134]
*L. plantarum*	Enhance the action of brain-derived neurotropic factor (BDNF), lowering the stress level in hippocampus neuron	[135]
*L. reuteri* DSM 17938	Modulate intestinal and colon motility and enhance gut–brain intercommunication for CNS homeostasis	[136]
*L. rhamnosus*	Reduce the behavioral changes including anxiety and depression	[137]

**Table 5:** BBB-breaching pathogenic infections and associated immunomodulatory activity.

Pathogen	Mode of Pathogenesis	Immunological Factors Contributing BBB Infection	References
*A. baumannii*	-Meningitis -Catheter-associated infection	Increased inflammatory cell response, toll-like receptor (TLR) altered expression, and proinflammatory cytokine burst within 24 h of infection	[142,143]
*E. coli*	-Endothelial cells -Attenuation of transforming growth factor (TGF)-β 1 signaling	Increased expression of endothelial-derived platelet-derived growth factor receptor (PDFGR) and intercellular adhesion molecule (ICAM), resulting in inflammation	[144]
*H. influenzae*	-Large amount of vascular endothelial growth factor receptor (VEGFR) -Adenosine receptor dysfunction	Endothelial disruption and tight junction altered expression: downregulation of tumor necrosis factor (TNF-α); endothelial proliferation	[145,146]
*K. pneumoniae*	-Cerebrospinal infection -Intracranial infection	Increased production of proinflammatory cytokines and chemokines; induction of hypoxia inducible factor (HIF)-1α	[147]
*L. monocytogenes*	-Vimentin-mediated infection Neuroinflammation	In1F virulent factor-associated downregulation of tight junction and overexpression of PDFGR and ICAM, resulting in inflammation	[148]
*N. meningitidis*	-Secretion of IgA protease -Evasion of immune response	Deformation of adherence junction, triggering IL-6 and IL-8 expression: leukocyte infiltration and infected phagocyte movement	[149,150]
*P. aeruginosa*	-Cerebrospinal infection -Meningitis	Increased production of inflammatory cell response; overproduction of IL-1β and IL-6	[151]
*S. aureus*	-Brain abscesses and endocarditis -Cytokine burst	Stimulate immune invasion, T cell activation: burst of proinflammatory cytokines; TNF-α, IL-6, and IL-10 overproduction	[152]
*S. pneumoniae*	-Neonatal meningitis -Laminin receptor transcytosis	TNF-α, IL-6, and IL-10 overproduction and increased permeability through anchored tight junction; cleavage of IgA through *pneumococci* IgA protease	[153,154]

**Table 6:** Probiotic-derived bEVs against BBB-invading pathogens.

Origin of bEV	Physiological Roles	Invading Pathogen(s)	References
*Burkholderia thailandensis* with quinolone	Synergistic antibiofilm activity	*Streptococcus* spp.	[158]
*E. coli* Nissle 1917	Increased anti-inflammatory properties, such as IL-10 and T helper (T_H_) cell-mediated cytotoxicity	*E. coli* and *S. aureus*	[159]
*L. crispatus* and *L. jensenii*	Antibiofilm and anti-inflammatory effect	*Candida albicans*	[160]
*L. paracasei* and *L. plantarum*	Decrease pro-inflammatory cytokine production	Enteroinvasive *E. coli*	[161]

## Data Availability

Not applicable.
